# Dose-Informed Surgical Margin in Mandibular Osteoradionecrosis Surgery: Integrating Radiation Biology into Osteotomy Planning

**DOI:** 10.3390/clinpract16100194

**Published:** 2026-10-19

**Authors:** Efsun Somay, Erkan Topkan, Ugur Selek

**Affiliations:** 1 Department of Oral and Maxillofacial Surgery, Faculty of Dentistry, Baskent University, 06597 Ankara, Turkey; 2 Department of Radiation Oncology, Faculty of Medicine, Baskent University, 01120 Adana, Turkey; docdretopkan@gmail.com; 3 Department of Radiation Oncology, School of Medicine, Koc University, 34650 Istanbul, Turkey; ugurselek@yahoo.com

**Keywords:** mandibular osteoradionecrosis, surgical margin, radiation dosimetry, virtual surgical planning, mandibular reconstruction, dose-informed surgical margin (DISM)

## Abstract

Mandibular osteoradionecrosis (ORN) remains one of the most challenging late complications of head and neck radiotherapy. Although surgical resection with vascularized reconstruction is the standard treatment for advanced disease, determination of the osteotomy margin continues to rely largely on anatomical findings, intraoperative assessment, and surgical judgment. However, accumulating evidence indicates that radiation-induced mandibular injury is spatially heterogeneous and biologically dynamic. Consequently, visible necrosis, geometric distance from disease, and punctate marrow bleeding may not reliably reflect the biological competence of residual irradiated bone. This review proposes the Dose-Informed Surgical Margin (DISM) framework, a biologically informed, hypothesis-generating concept that integrates patient-specific historical radiotherapy dosimetry with conventional anatomical, radiological, intraoperative, and host-related factors to support individualized osteotomy planning. We examine the radiobiological basis of mandibular injury, current concepts of surgical margin assessment, the limitations of bleeding-guided resection, and the potential contribution of three-dimensional radiation dose mapping to identifying biologically more favorable residual bone. We further discuss technical considerations for clinical implementation, including image registration, multimodality imaging, virtual surgical planning, and integration with host-related determinants of tissue repair. Rather than advocating universal dose thresholds or replacing established surgical principles, DISM is intended as an adjunctive decision-support framework that adds biological context to conventional margin assessment. Prospective biological and clinical validation remains essential before routine clinical implementation. If validated, DISM may facilitate a transition from predominantly anatomical definitions of mandibular osteotomy toward biologically informed, patient-specific surgical planning for the management of mandibular ORN.

## 1. Introduction

Mandibular osteoradionecrosis (ORN) remains one of the most debilitating late complications of radiotherapy (RT) for head and neck cancer [1,2,3,4]. Although contemporary techniques, including intensity-modulated RT (IMRT), volumetric-modulated arc therapy (VMAT), and proton therapy, have improved dose conformality and reduced exposure of uninvolved tissues, clinically significant mandibular irradiation often remains unavoidable when target volumes lie adjacent to, overlap with, or involve the mandible [3,4,5,6,7,8]. The reported incidence of ORN ranges from approximately 5% to 15%, with substantial variation according to tumor site, treatment era, mandibular dose-volume exposure, dental status, dentoalveolar trauma, and host-related factors [1,2,3,4,5,6,7,8]. Once established, ORN may cause persistent pain, infection, pathological fracture, orocutaneous fistula, impaired mastication, and marked deterioration in quality of life, in some cases necessitating mandibular resection and vascularized free-tissue reconstruction [1,2,3,4,9,10,11,12,13].

Management of mandibular ORN ranges from conservative treatment in early-stage disease to segmental mandibulectomy with vascularized free-flap reconstruction in advanced cases. International guidelines recommend removal of necrotic bone while preserving functional mandibular tissue whenever feasible. However, the optimal extent of mandibular resection remains insufficiently defined. Unlike oncologic surgery, in which histopathological principles guide margin assessment, ORN lacks a widely accepted biological standard for defining a safe resection margin. Furthermore, ORN progression has been reported despite apparently adequate resection to bleeding bone and vascularized free-flap reconstruction; in the series by Zaghi et al., persistent disease occurred in 23.5% of patients despite extensive mandibular resection to bleeding bone, and progression was not explained by histologically necrotic margins [14]. Histopathological studies have also demonstrated considerable biological heterogeneity at mandibular resection margins, further underscoring the limitations of current intraoperative margin assessment [15,16]. Consequently, osteotomy planning still relies primarily on preoperative imaging, intraoperative assessment of bone vitality, punctate bleeding, and surgical judgment rather than objective radiobiological criteria [17,18,19,20].

This gap is clinically significant because radiation-induced mandibular injury is unlikely to terminate abruptly at the visible margin of necrosis [1,21,22]. Rather, radiation produces a spatially heterogeneous continuum of tissue changes, including endothelial injury, microvascular rarefaction, hypocellularity, fibroatrophy, chronic inflammation, impaired osteogenesis, and reduced regenerative capacity [1,21,22,23,24,25]. Consequently, mandibular bone that appears macroscopically intact may remain biologically compromised within the irradiated field, with potential implications for healing after resection or reconstruction [1,21,22]. Histopathological evidence of heterogeneous necrotic and reparative changes at mandibular resection margins further supports the concern that macroscopic appearance alone may not reliably define biologically competent residual bone [15].

In this review, biological competence is used as a conceptual, multidimensional construct referring to the capacity of residual irradiated mandibular bone to remain viable and support durable healing after osteotomy and reconstruction. It encompasses related but non-equivalent domains, including perfusion and microvascular integrity, cellular viability, bone-remodeling activity, and regenerative capacity [1,21,22,23,24,25]. For this framework, no single clinical, imaging, histopathological, or dosimetric measure directly quantifies biological competence. Postoperative outcomes such as wound healing, osseous union, recurrent or progressive ORN, fistula, infection, plate exposure, and revision surgery are regarded as downstream clinical manifestations that may reflect, but do not individually define, this construct.

In the past decade, several dosimetric studies have reported associations between mandibular dose-volume parameters—including mean dose, maximum dose, and volume-based thresholds such as V30, V40, V50, V60, and V70—and the risk of ORN development [3,5,6,7,8]. These studies address susceptibility to developing ORN after RT and have contributed to risk stratification and efforts to minimize mandibular exposure during treatment planning [3,5,6,7,8]. They do not, however, establish that historical radiation dose at a proposed osteotomy site predicts postoperative healing or recurrence once ORN has developed. Evidence for this latter application is substantially more limited. Preliminary surgical-planning studies suggest that incorporation of isodose information is technically feasible and may help contextualize margin selection, although robust outcome-based evidence remains limited [26,27].

Advances in virtual surgical planning and computer-assisted reconstruction have enabled three-dimensional visualization of previous mandibular RT isodose distributions during surgical planning. Early clinical studies have demonstrated the technical feasibility of integrating patient-specific isodose information into mandibular ORN surgery and provided preliminary observations linking the radiation environment of residual bone with postoperative outcome [26,27]. In particular, recurrence has been reported when mandibular resection margins remained within high-dose isodose volumes, supporting further investigation of the potential clinical relevance of local radiation exposure. The proposed DISM does not claim novelty for isodose visualization itself. Rather, its contribution is to extend these earlier technical and exploratory approaches into a multidimensional conceptual framework in which historical radiation dose is interpreted as an adjunctive spatial exposure metric and integrated with anatomical and radiological disease extent, intraoperative viability assessment, host-related vulnerability, and reconstructive considerations. DISM further distinguishes prior radiation exposure from current biological competence and frames the resulting construct as a hypothesis-generating decision-support approach requiring biological and clinical validation rather than as a dose-based surgical protocol.

This conceptual review therefore introduces the Dose-Informed Surgical Margin (DISM) as a hypothesis-generating framework for mandibular ORN surgery. Rather than treating millimetric distance, radiological bone destruction, intraoperative bleeding, surgical judgment, or historical radiation dose as independently sufficient criteria, DISM conceptualizes the osteotomy margin as an interface whose suitability is informed by the combined anatomical, radiological, intraoperative, dosimetric, and patient-related context. To develop this framework, the review synthesizes evidence from radiobiology, mandibular dosimetry, histopathology, imaging, and surgical management; examines current concepts of surgical-margin assessment; identifies the biological limitations of conventional millimeter-based and bleeding-based approaches; and proposes DISM as a foundation for future translational investigation and prospective biological and clinical validation.

## 2. Review Type and Literature Selection

This article is presented as a conceptual, hypothesis-generating review rather than a systematic review or meta-analysis. It does not aim to provide exhaustive evidence synthesis, but to critically integrate findings from radiobiology, radiation dosimetry, histopathology, imaging, and surgical management that are relevant to mandibular ORN and the proposed DISM framework.

The literature was identified through iterative searches of PubMed/MEDLINE from database inception through [20 August 2026], supplemented by examination of the reference lists of relevant articles and key reviews. Representative search concepts included osteoradionecrosis, mandible or mandibular, radiotherapy, radiation dose, dosimetry, isodose, surgical margin, osteotomy, mandibular resection, virtual surgical planning, histopathology, bone viability, perfusion or bleeding bone, and mandibular reconstruction. Search terms were combined according to the topic being examined rather than through a single prespecified Boolean search string, consistent with the conceptual nature of the review.

Literature selection prioritized international clinical practice guidelines, consensus statements, systematic reviews, key experimental and histopathological studies, dosimetric investigations of mandibular ORN risk, studies of surgical management and reconstruction, and reports directly addressing incorporation of patient-specific RT dose information into mandibular ORN surgical planning. Studies were selected according to their relevance to the biological, technical, or clinical questions addressed by the framework rather than through predefined systematic-review eligibility criteria. Particular attention was given to evidence distinguishing associations between mandibular radiation exposure and ORN development from the more limited evidence relating local osteotomy-site dose to postoperative healing or recurrence.

Because the review was designed for conceptual synthesis rather than quantitative evidence aggregation, no formal risk-of-bias assessment, study-quality scoring system, meta-analysis, or PRISMA-style study-selection process was undertaken. Areas in which the evidence is indirect, preliminary, or derived from small feasibility studies are identified explicitly in the relevant sections and tables.

## 3. Why Conventional Surgical Margins May Be Biologically Inadequate

Conventional mandibular ORN surgery relies primarily on anatomical and intraoperative indicators of disease extent and bone viability. While these criteria remain clinically indispensable, they may not fully reflect the spatially heterogeneous and progressive nature of radiation-induced bone injury. This section examines why conventional margin assessment may be anatomically reasonable yet biologically incomplete, thereby providing the rationale for a dose-informed approach.

### 3.1. Current Margin Assessment in Mandibular ORN

Surgical management of advanced mandibular ORN focuses on excision of necrotic bone while preserving as much functional mandibular tissue as possible. The objective is to remove nonviable bone without unnecessarily compromising mandibular continuity, occlusion, speech, swallowing function, or reconstructive options. In current practice, osteotomy site selection typically relies on preoperative imaging, intraoperative visual and tactile assessment, and observation of punctate marrow bleeding, which has historically been regarded as a practical surrogate marker of residual bone viability [9,10,11,12].

Although widely adopted in clinical practice, this approach lacks a robust biological foundation. In oncologic surgery, margin assessment is supported by established histopathological criteria for microscopic tumor spread; by contrast, mandibular ORN has no analogous biological standard for determining the extent of bone resection. International guidelines recommend removing necrotic bone but do not specify objective criteria to distinguish irradiated but viable bone from biologically compromised residual tissue [2,5,13]. Importantly, neither current guidelines nor commonly cited surgical series define a universally accepted millimetric safety margin for mandibular ORN resection; instead, margin selection generally reflects radiological disease extent, macroscopic assessment, punctate bleeding, and surgeon judgment. As a result, the definition of a “safe” surgical margin in ORN remains primarily informed by imaging findings, intraoperative assessment, and surgeon judgment rather than validated radiobiological, histopathological, or molecular criteria, despite evidence that ORN may progress after resection to bleeding bone and free-flap reconstruction [14].

### 3.2. Limitations of Punctate Bleeding as a Viability Marker

The conventional use of punctate marrow bleeding as an indicator of bone viability highlights an important limitation of current surgical margin assessment in mandibular ORN. Intraoperative bleeding suggests residual perfusion, but it does not necessarily confirm preserved microvascular integrity, osteocyte survival, osteoblast function, mesenchymal stem-cell activity, or regenerative potential. Similarly, cortical continuity and gross bone appearance provide primarily anatomical information and may not adequately reflect the cumulative biological effects of radiation injury [9,10,11,12,13].

This distinction is clinically important because ORN is increasingly understood as a progressive radiation-induced tissue injury rather than a strictly localized area of exposed or necrotic bone. Radiation injury evolves along a continuum involving endothelial dysfunction, microvascular obliteration, hypocellularity, fibroatrophy, impaired osteoblast activity, reduced bone remodeling, and chronic inflammation [18,21,23,24,25]. Histopathological evidence of heterogeneous necrotic and reparative changes at mandibular resection margins further supports the concern that macroscopically viable bone may not always be biologically competent [15]. Accordingly, punctate bleeding should be regarded as evidence of residual perfusion rather than as a comprehensive measure of the biological condition or healing potential of irradiated bone.

### 3.3. Clinical Evidence Suggesting Margin Vulnerability

Clinical outcomes following advanced mandibular ORN surgery illustrate that postoperative healing and disease control cannot be predicted from intraoperative margin appearance alone. Complications—including delayed wound healing, wound dehiscence, plate exposure, persistent fistula, recurrent infection, and disease progression—have been reported despite segmental resection and vascularized reconstruction [14,28,29,30,31]. These outcomes are inherently multifactorial and may reflect disease severity, infection, soft-tissue condition, fixation and reconstructive factors, host-related vulnerability, and characteristics of the residual irradiated bone. Their occurrence therefore should not be attributed solely to the adequacy or inadequacy of bleeding-guided margin selection.

Studies incorporating virtual surgical planning and RT isodose visualization further highlight the absence of standardized, biologically based criteria for osteotomy planning. In particular, ORN progression despite resection to bleeding bone and vascularized free-tissue reconstruction indicates that the presence of punctate bleeding at the osteotomy margin does not by itself guarantee durable postoperative disease control [14,26,27,28,29,30,31]. Moreover, histopathological analyses have demonstrated heterogeneous patterns of necrosis and tissue repair at mandibular resection margins, indicating that macroscopic appearance alone does not sharply delineate the transition between nonviable and recoverable bone [15]. Together, these observations support interpreting intraoperative margin findings within a broader multifactorial context rather than regarding them as independent determinants of postoperative outcome.

### 3.4. Why Millimetric Distance Does Not Equal Biological Safety

A key limitation of conventional margin assessment is the assumption that a fixed anatomical distance from visible necrosis ensures biological safety. However, geometric distance alone is unlikely to determine the biological status of irradiated bone; cumulative radiation exposure, local dose heterogeneity, and the downstream vascular, cellular, and remodeling effects of radiation injury also influence it [23,24,25,32,33,34].

For instance, a 10-mm osteotomy margin may correspond to mandibular bone receiving less than 40 Gy in one patient, whereas an anatomically similar margin in another case may remain within a 60–70 Gy isodose region. Although these margins are geometrically equivalent, they may not be biologically equivalent, because local radiation dose can influence vascular integrity, cellularity, bone remodeling, and healing potential [23,24,25,26,27,32,33,34,35,36].

### 3.5. Dose Heterogeneity as the Rationale for DISM

Recent three-dimensional dosimetric studies have questioned whether anatomical distance alone is sufficient as the sole criterion for surgical-margin assessment in mandibular ORN. Integration of RT isodose visualization into surgical planning enables characterization of the dose environment of the residual mandible, which may influence postoperative outcomes. In particular, recurrence or progression after segmental resection has been reported when residual mandibular bone remained within high-dose isodose volumes, supporting the premise that radiation dose distribution may convey clinically relevant information beyond anatomical assessment alone [26,27,32,37].

These findings suggest that traditional millimetric margins, although anatomically defined, may not always correspond to biologically adequate resection in the context of heterogeneous radiation dose distributions. Emerging clinical and histopathological evidence supports conceptualizing the osteotomy line as a biological interface between irradiated bone with preserved reparative capacity and bone at heightened risk of progressive injury. The DISM framework reflects this shift by incorporating patient-specific radiation dose information alongside clinical, radiological, and intraoperative data to identify residual mandibular segments with the greatest potential for durable healing.

## 4. Radiotherapy Dosimetry as a Surgical Exposure Metric

Radiotherapy dosimetry has traditionally informed estimates of treatment-related toxicity and guided planning to minimize complications [3,5,6,7,8,32,33,34,35,36]. In mandibular ORN, numerous clinical and dosimetric studies have consistently reported associations between higher mandibular dose-volume exposure and increased risk of ORN development [3,5,6,7,8,32,33,34,35,36]. However, the application of patient-specific dosimetric data to surgical decision-making after ORN onset remains insufficiently studied. Recent reports using three-dimensional dose mapping and postoperative outcome assessment suggest that local radiation dose may influence healing potential and recurrence risk following mandibular resection [26,27,32,37]. This section reviews the rationale for interpreting radiation dose distribution as a spatial exposure metric of mandibular tissue injury and discusses its emerging role in surgical-margin assessment.

### 4.1. Mandibular Dose–Volume Parameters and the Risk of Osteoradionecrosis

Over the past two decades, a substantial body of evidence has identified mandibular radiation dose as one of the most consistent predictors of ORN following RT for head and neck cancer. Retrospective cohort studies, systematic reviews, and dose–response analyses have consistently reported significant associations between mandibular dose–volume parameters and ORN risk [3,5,6,7,8,32,33,34,35,36,38,39,40], reinforcing the central role of radiation dosimetry in toxicity prediction and RT treatment planning.

Among the dosimetric variables evaluated, mean mandibular dose, maximum dose, and volume-based parameters—including V30, V40, V50, V60, and V70—have demonstrated reproducible associations with ORN risk across diverse patient populations and RT techniques [5,6,7,8,32,33,34,35,36,39,40]. Although the relative predictive performance of individual parameters varies among studies because of differences in patient populations, contouring methodology, RT technique, and ORN definition, a consistent finding is that greater mandibular radiation exposure is associated with an increased likelihood of radiation-induced bone injury.

These observations have substantially influenced contemporary RT practice. Modern IMRT, VMAT, and proton therapy routinely incorporate mandibular dose constraints during treatment planning to reduce ORN risk whenever oncologically feasible [3,5,6,7,8]. Consequently, mandibular dosimetry has become an integral component of ORN risk assessment and prevention and represents one of the most extensively studied quantitative exposure measures associated with ORN development. Importantly, however, this evidence concerns the risk of developing ORN and does not directly validate local radiation dose at an osteotomy site as a predictor of postoperative healing, persistent disease, or recurrence after surgical treatment of established ORN.

Despite this robust body of evidence, the clinical application of mandibular dosimetry has remained largely confined to predicting ORN before its onset. Comparatively little attention has focused on whether the same patient-specific dose information may also inform surgical decision-making after ORN has developed. This translational gap supports investigating whether patient-specific radiation dose distribution, interpreted as a spatial record of prior exposure, may provide additional information for biologically informed mandibular osteotomy planning.

### 4.2. From ORN Risk Prediction to Surgical Decision-Making

The well-documented association between mandibular radiation dose and the development of ORN provides a biological rationale—but not direct clinical validation—for investigating whether patient-specific historical dose may also inform surgical management after ORN has developed. These represent distinct evidentiary questions: the former concerns the probability of ORN occurrence after irradiation, whereas the latter concerns healing and disease control in residual mandibular bone at or adjacent to a selected osteotomy site. Evidence addressing the second question remains limited [26,27,32,37].

From a radiobiological perspective, this extension is plausible because radiation dose contributes to endothelial injury, microvascular rarefaction, fibroatrophy, hypocellularity, impaired bone remodeling, and reduced regenerative capacity within irradiated bone [18,21,23,24,25]. These changes are spatially heterogeneous and likely vary with local radiation dose distribution. Thus, residual mandibular segments with similar anatomical appearance may differ in postoperative healing capacity depending on their cumulative radiation exposure.

This distinction suggests that mandibular dosimetry may provide clinically relevant information beyond prediction of ORN occurrence. Patient-specific dose distribution provides an objective spatial record of prior radiation exposure and may therefore offer indirect information relevant to cumulative tissue injury, while not directly measuring current perfusion, cellular viability, remodeling activity, or regenerative capacity. Within this paradigm, radiation dose is one component of a broader assessment of the residual mandible rather than a direct measure of its biological competence.

Accordingly, the potential role of mandibular dosimetry shifts from prevention alone toward biologically informed surgical planning. Although this approach has not yet been validated prospectively, it provides the theoretical basis for incorporating patient-specific historical dose distribution as an adjunctive exposure metric within the DISM framework.

### 4.3. Isodose-Informed Surgical Planning: Current Clinical Evidence

In contrast to the extensive literature relating mandibular radiation dose to the development of ORN, direct evidence linking radiation dose at or near an osteotomy site with postoperative healing, persistent disease, or recurrence remains sparse. Existing studies have primarily demonstrated the technical feasibility of integrating three-dimensional RT dose distributions into virtual surgical planning and incorporating patient-specific isodose information into mandibular ORN surgery [26,27,32,37]. These early reports establish technical feasibility and suggest that dosimetric information may provide clinically relevant spatial context for operative decision-making, but they do not validate dose-based osteotomy criteria or establish a causal relationship between osteotomy-site dose and postoperative outcome.

Superimposing RT dose maps onto three-dimensional mandibular models enables visualization of the spatial relationship between planned osteotomy sites and the local radiation environment. This method extends conventional anatomical planning by incorporating cumulative radiation exposure of the residual mandible, allowing assessment of surgical margins within a dosimetric framework rather than solely by geometric proximity to visible necrosis [26,27].

Preliminary clinical observations suggest that residual mandibular bone within higher-dose isodose regions may be associated with less favorable postoperative outcomes, including persistent or recurrent ORN. Although these findings are derived mainly from small feasibility studies and retrospective clinical series, they provide hypothesis-generating evidence that local radiation exposure may be associated with postoperative outcomes after mandibular ORN surgery [26,27,32,37].

These studies do not establish universal dose thresholds for mandibular osteotomy or validate radiation dose as an independent predictor of postoperative outcome. Rather, they demonstrate the feasibility of integrating patient-specific RT dosimetry into surgical planning and provide preliminary evidence supporting further investigation of its potential clinical and biological relevance. Further prospective studies with standardized dosimetric protocols, histopathological validation, and long-term follow-up are necessary before dose-informed osteotomy planning can be adopted in routine practice.

### 4.4. Interpreting Radiation Dose as a Continuous Biological Risk Gradient

A potential misconception in applying RT dosimetry to mandibular osteotomy planning is assuming that a single radiation dose threshold defines a universally safe surgical margin. However, current evidence does not support a discrete dose cutoff. Radiobiological and clinical investigations suggest that radiation-induced mandibular injury occurs along a continuum, with incremental deterioration in vascular integrity, cellularity, bone remodeling, and regenerative capacity as cumulative radiation dose increases [18,21,23,24,25,32,33,34,35,36].

Accordingly, radiation dose is best regarded as a continuous biological risk gradient rather than a binary determinant of tissue viability. Bone exposed to intermediate doses may retain meaningful reparative capacity, whereas regions receiving higher doses may exhibit greater biological compromise. The transition from biologically competent to biologically vulnerable bone is unlikely to align with a single isodose line; instead, it reflects a spectrum of injury shaped by local radiation exposure, tissue characteristics, and host susceptibility [18,21,23,24,25,32,33,34,35,36].

Moreover, radiation dose alone does not fully determine postoperative healing. Patient-specific factors, including vascular status, nutritional and inflammatory condition, smoking history, dental health, comorbidities, and prior dentoalveolar trauma, as well as treatment-related variables such as radiation technique, fractionation, and concomitant systemic therapy, can all influence tissue resilience and reparative capacity [1,2,3,4,18,23,24,25]. Therefore, identical radiation doses may yield divergent biological effects across individuals.

Within the DISM framework, radiation dosimetry is considered one component of a multidimensional biological assessment rather than an isolated determinant of surgical decision-making. Its primary value lies in providing objective, patient-specific information on the spatial distribution of radiation exposure, which can complement anatomical, radiological, and intraoperative evaluation when selecting biologically appropriate mandibular osteotomy margins [26,27]. This approach supports individualized surgical planning while avoiding reliance on dose thresholds that have not been prospectively validated.

## 5. Biological Basis of the DISM

An evolving understanding of the biological mechanisms by which ionizing radiation alters the vascular, cellular, and regenerative properties of mandibular bone supports the rationale for the DISM. Experimental, histopathological, imaging, and clinical studies suggest that radiation-induced injury is spatially heterogeneous, temporally progressive, and influenced by both local dose exposure and individual patient factors [15,18,21,23,24,25,32,33,34,35,36]. These findings provide a mechanistic rationale for considering patient-specific radiation dose distribution as a spatial exposure metric that may contribute indirect information about residual tissue condition, while recognizing that historical dose does not directly measure current biological competence. Within the DISM framework, dosimetric information is therefore interpreted as complementary to anatomical, radiological, and intraoperative assessments in surgical planning.

### 5.1. Spatial Heterogeneity of Dose-Dependent Radiation Injury

Radiation-induced injury of the mandible is spatially heterogeneous because radiation dose is distributed nonuniformly within irradiated bone. Contemporary RT modalities, including IMRT, VMAT, and proton therapy, generate complex three-dimensional dose distributions with high-dose regions, intermediate-dose gradients, and relatively spared zones within the same mandibular segment [3,5,6,7,8,26,27,32,33,34,35,36]. As a result, adjacent regions of mandibular bone may receive substantially different cumulative doses despite close anatomical proximity.

Experimental, radiobiological, and clinical investigations suggest that spatial heterogeneity in radiation exposure is accompanied by heterogeneity in biological response. Progressive endothelial injury, microvascular rarefaction, fibroatrophy, hypocellularity, impaired osteoblast function, diminished bone remodeling, and reduced regenerative capacity have been associated with increasing radiation exposure, although the magnitude and temporal evolution of these changes may differ among irradiated tissues [18,21,23,24,25,32,33,34,35,36]. These findings support the view that the biological status of irradiated mandibular bone exists along a continuum rather than as a uniform tissue state.

This concept has important implications for surgical planning. If biological injury varies spatially according to local dose distribution, anatomical distance alone may not reliably distinguish bone with sufficient reparative potential from tissue at greater risk of compromised healing. Patient-specific radiation dose maps may therefore provide additional information regarding the spatial heterogeneity of tissue injury, supplementing anatomical, radiological, and intraoperative assessment during mandibular osteotomy planning [26,27].

### 5.2. The Biological Penumbra of Irradiated Bone: A Hypothesis-Generating Analogy

Conventional mandibular osteotomy planning often presumes that the visible or radiographically defined boundary of ORN closely approximates the transition from nonviable to viable bone. However, experimental, histopathological, imaging, and clinical evidence suggests that radiation-induced tissue injury may extend beyond the macroscopically apparent limits of necrosis [15,18,21,23,24,25]. These findings suggest that the biological effects of irradiation are not restricted to visibly affected bone but may also involve adjacent mandibular regions with preserved structural integrity.

A putative transitional region beyond overt necrosis may contain irradiated bone that maintains gross anatomical integrity while exhibiting varying degrees of endothelial dysfunction, microvascular rarefaction, hypocellularity, fibroatrophy, impaired bone remodeling, and reduced regenerative capacity [9,15,18,21,22,23,24,25,26]. Histopathological studies further suggest that cortical and cancellous bone may exhibit heterogeneous patterns of residual viability at surgical margins despite similar macroscopic appearance, supporting the concept of spatially variable biological competence [15,41]. These observations provide a biological rationale for hypothesizing such a transitional region but do not establish its existence, spatial boundaries, or pathological definition.

In this review, “biological penumbra” is used as a hypothesis-generating analogy for a putative transitional region beyond grossly necrotic tissue, where biological impairment may precede overt structural failure. The term is conceptually analogous to a penumbra as a transition rather than a sharply demarcated boundary, but it is not used here in the conventional radiotherapy-physics sense of a beam-edge dose-gradient region. Nor is it intended to denote an established histopathological entity, a clinically delineable zone, or a region defined by a specific radiation-dose range. Rather, the concept highlights the hypothesis that radiation-related biological injury may extend beyond the macroscopically apparent boundaries of ORN [15,18,21,23,24,25].

This biological-penumbra hypothesis provides a conceptual rationale for the DISM. Rather than assuming the visible edge of necrosis defines the biological boundary of disease, DISM proposes that patient-specific radiation dose distribution may provide spatial exposure context relevant to potential differences in residual tissue condition and healing capacity, complementing anatomical, radiological, and intraoperative assessment during osteotomy planning [25,26].

### 5.3. Interaction Between Radiation Dose and Host Tissue Vulnerability

Radiation dose alone does not fully account for the development, progression, or postoperative behavior of mandibular ORN. Although local dose exposure is associated with endothelial injury, microvascular compromise, fibroatrophy, hypocellularity, and impaired bone remodeling, patient-specific host factors also modify radiation’s biological impact [1,2,3,4,18,21,23,24,25].

Host-related variables, including smoking, diabetes mellitus, vascular status, anemia, nutritional reserve, systemic inflammation, oral hygiene, periodontal disease, and prior dentoalveolar trauma, may influence tissue oxygenation, immune regulation, wound healing, and skeletal repair [1,2,3,4,6,13,29]. Thus, patients with similar mandibular dose distributions may differ considerably in residual bone competence and postoperative healing capacity because of differences in baseline tissue vulnerability.

This interaction between dose exposure and host vulnerability is central to the DISM framework. Radiation dose should be interpreted as one component of a broader biological context that includes local dosimetry, systemic repair capacity, dental status, and reconstructive considerations. DISM is therefore best understood as a personalized surgical-planning framework that integrates multiple dimensions of patient and tissue biology rather than dosimetric parameters alone.

A conceptually analogous observation has recently been reported in medication-related osteonecrosis of the jaw (MRONJ). Soldatos et al. described stage 2 MRONJ developing after dentoalveolar surgery and implant placement despite an 11-year interval since cessation of high-dose intravenous bisphosphonate therapy and a serum C-terminal telopeptide of type I collagen (CTX) value that might historically have been interpreted as reassuring [42]. Although MRONJ and ORN are etiologically distinct entities and this observation cannot be extrapolated directly to radiation-induced bone injury, the case illustrates a broader principle relevant to the present framework: persistent treatment-related tissue vulnerability may not be reliably captured by a single contemporary clinical or biochemical surrogate. In ORN, this analogy further supports multidimensional assessment rather than reliance on any individual marker of tissue competence.

### 5.4. DISM as an Integrated Biological Framework

The DISM synthesizes current biological concepts into a unified framework for mandibular osteotomy planning. The underlying principle is that the biological suitability of a resection margin is not reliably determined by anatomical appearance, millimetric distance, or intraoperative bleeding alone. Instead, margin adequacy should be evaluated through an integrative approach that considers the extent of visible disease, radiological bone destruction, intraoperative viability indicators, patient-specific radiation dose distribution, and host tissue vulnerability [15,23,24,25,26,27]. Table 1 summarizes the principal evidence domains relevant to the DISM framework and their corresponding evidentiary gaps.

Within this framework, RT dosimetry provides a spatial record of cumulative radiation exposure. Dose information is not intended to replace clinical judgment, imaging interpretation, or intraoperative assessment; rather, it adds a biological dimension to conventional planning by helping identify regions of the residual mandible with differing capacities for durable healing [26,27,32,33,34,35,36]. In this way, DISM conceptualizes the osteotomy line as a biologically informed interface between compromised and potentially recoverable irradiated bone, rather than as a strictly geometric boundary.

Accordingly, DISM is not a fixed-dose threshold model or an independent surgical protocol. It is best understood as a personalized, hypothesis-generating decision framework intended to inform future biologically guided mandibular reconstruction. Prospective studies are needed to evaluate whether DISM-guided planning improves wound healing, reduces recurrent ORN, and enhances long-term reconstructive outcomes.

## 6. Is Bleeding Bone Truly Healthy Bone? Reconsidering the Surgical Endpoint in Mandibular ORN

For decades, punctate marrow bleeding has served as a principal intraoperative indicator of residual bone viability during mandibular ORN surgery. Because of its simplicity and immediate observability, bleeding has often guided osteotomy placement and estimation of surgical-margin adequacy [9,10,11,12,13]. Although this criterion is widely accepted, the biological basis for equating intraoperative bleeding with long-term bone competence remains insufficiently substantiated. Reports of persistent ORN despite resection to bleeding bone illustrate that the presence of bleeding alone does not ensure durable postoperative disease control, although such outcomes are multifactorial and cannot be attributed solely to the margin-assessment method [14].

Emerging radiobiological, histopathological, and clinical evidence suggests that visible perfusion does not necessarily indicate preserved vascular integrity, cellular viability, or the regenerative capacity required for durable healing [15,18,21,23,24,25]. Because radiation-induced injury evolves spatially and temporally, irradiated bone may retain the ability to bleed intraoperatively while still harboring biological alterations relevant to healing. These observations support interpreting intraoperative bleeding as one component of a multifactorial biological assessment rather than as a definitive endpoint for surgical-margin selection.

Accordingly, this section examines the biological significance and limitations of punctate marrow bleeding, reviews its relationship to tissue competence, and discusses how the DISM framework may support a more integrative surgical endpoint by incorporating intraoperative findings, patient-specific radiation dosimetry, and additional biological determinants of tissue viability.

### 6.1. Punctate Bleeding as the Traditional Surgical Endpoint

Punctate marrow bleeding has historically been regarded as a principal intraoperative indicator of residual bone viability in mandibular ORN surgery. The rationale is that bleeding from cancellous bone signifies preservation of local perfusion and, by inference, the presence of viable tissue capable of supporting postoperative healing. Its ease of assessment during surgery, without specialized equipment, has made punctate bleeding a practical endpoint for osteotomy margin determination and contributed to its widespread adoption in surgical practice [9,10,11,12,13].

The widespread use of punctate bleeding as a surgical endpoint reflects both historical practice and the lack of validated alternative methods for intraoperative assessment of mandibular tissue viability. Current clinical guidelines recommend removal of necrotic bone but do not specify objective biological criteria for margin adequacy [2,13,17]. Thus, punctate bleeding functions less as a validated biological endpoint than as a pragmatic intraoperative surrogate in the absence of standardized millimetric or histopathological margin criteria. As a result, punctate bleeding has become a de facto endpoint for mandibular osteotomy, although direct evidence supporting its ability to predict long-term tissue competence or reconstructive outcomes remains limited. Notably, Zaghi et al. reported persistent ORN in 23.5% of patients despite extensive mandibular resection performed to bleeding bone and vascularized free-tissue reconstruction [14]. This observation demonstrates that bleeding at the resection margin is not, by itself, sufficient to predict durable postoperative outcome; however, it does not establish that the bleeding-guided margin was the cause of treatment failure.

While punctate bleeding provides valuable information about local perfusion, it primarily indicates residual blood flow at the time of surgery. Whether this observation reflects the broader biological capacity of irradiated bone for durable healing remains uncertain and forms the basis for the subsequent sections of this review.

### 6.2. Bleeding as Perfusion, Not Biological Competence

The presence of punctate marrow bleeding during mandibular osteotomy primarily indicates residual blood flow within the exposed cancellous bone. Although local perfusion is an essential prerequisite for tissue viability, it does not necessarily indicate preservation of microvascular integrity, osteocyte and osteoblast function, mesenchymal stem-cell activity, or the regenerative capacity required for durable postoperative healing [15,18,21,23,24,25]. Consequently, perfusion and biological competence should not be regarded as synonymous.

This distinction is particularly relevant in irradiated bone. Radiation-induced vascular injury is characterized not only by reductions in vessel density but also by progressive endothelial dysfunction, fibrosis, chronic inflammation, hypocellularity, and impaired bone remodeling. These biological alterations may persist despite residual blood flow [15,18,21,23,24,25]. Accordingly, a mandibular segment that demonstrates punctate bleeding intraoperatively may still harbor radiation-related biological impairment. The degree of such impairment, however, cannot be inferred from bleeding status or radiation dose alone.

Histopathological studies demonstrate that viable and nonviable cortical and cancellous bone may coexist within the same mandibular specimen and that pathological characteristics at resection margins are associated with clinical outcomes, indicating that structural preservation and residual perfusion do not necessarily correspond to complete biological recovery of irradiated mandibular bone [15,40,41]. Therefore, while punctate bleeding remains a useful indicator of local perfusion, it should be interpreted as one component of tissue assessment rather than as definitive evidence of long-term biological competence. The occurrence of ORN progression after bleeding-guided resection further demonstrates that bleeding alone does not predict postoperative outcome, while not establishing that the margin-assessment method itself caused treatment failure [14].

**Figure 1:**
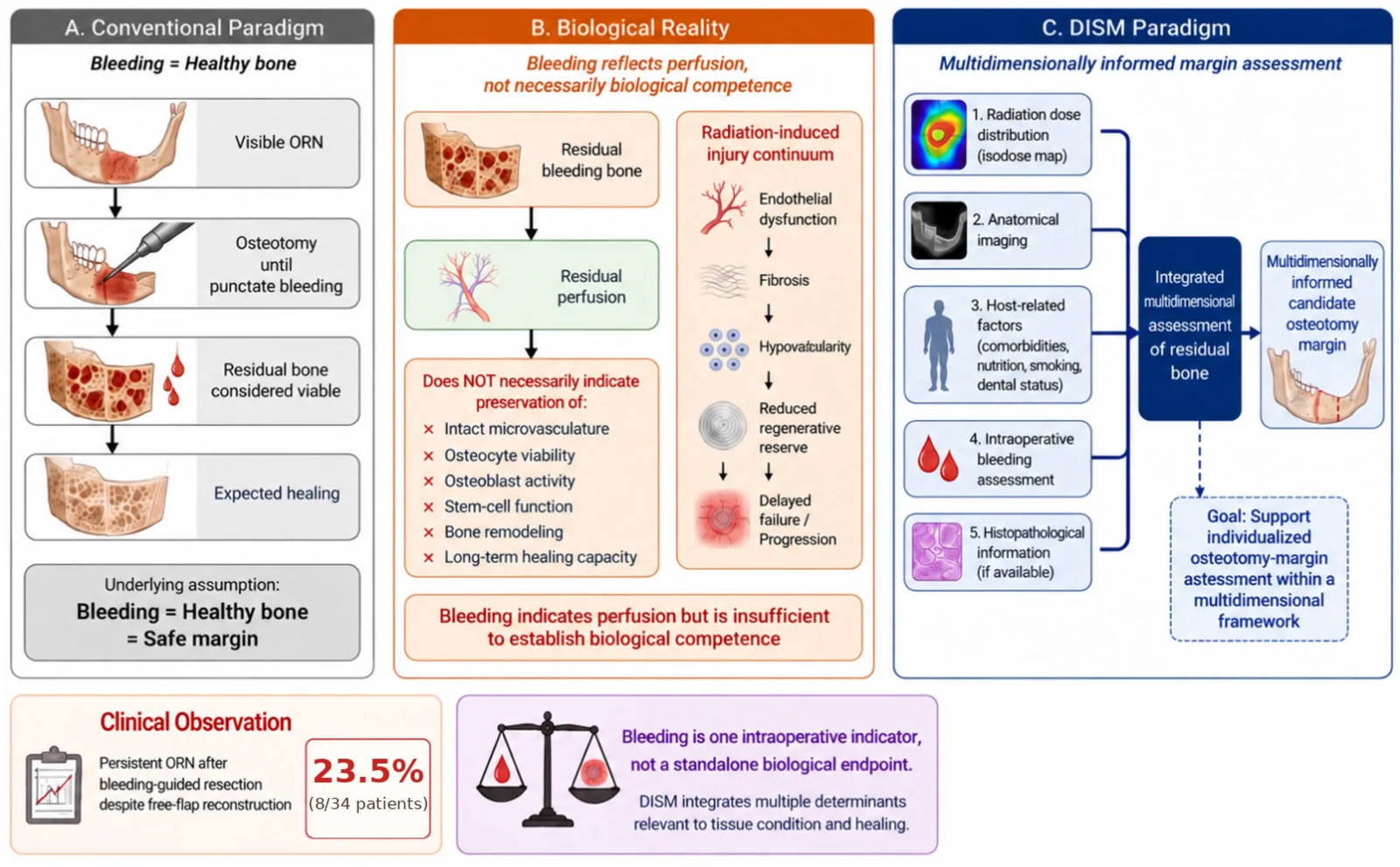
From Punctate Bleeding to Biological Competence: The Conceptual Basis of the DISM Framework. Punctate marrow bleeding primarily reflects residual perfusion and should not be interpreted as a direct measure of biological competence or as an independent predictor of postoperative outcome in irradiated mandibular bone. The DISM framework proposes interpreting bleeding together with patient-specific radiation dose distribution, anatomical imaging, host-related factors, reconstructive considerations, and histopathological information, when available, to support multidimensional osteotomy-margin assessment. The reported rate of persistent ORN after bleeding-guided resection (23.5%; 8/34 patients) is from Zaghi et al. [14].

These observations provide a biological rationale for integrating intraoperative findings with patient-specific RT dosimetry. Rather than replacing punctate bleeding as an intraoperative indicator, the DISM framework proposes interpreting bleeding within the broader context of prior radiation exposure and other anatomical, radiological, biological, host-related, and reconstructive factors relevant to tissue condition and healing [26,27]. Figure 1 illustrates the conceptual distinction between punctate bleeding, residual perfusion, and biological competence.

### 6.3. High-Dose Bleeding Bone and the Possibility of Delayed Failure

A potential limitation of bleeding-guided margin assessment is that irradiated bone may retain sufficient residual perfusion to bleed intraoperatively while still harboring biological alterations relevant to subsequent healing. This possibility may warrant particular consideration when the osteotomy line lies within a high-dose radiation region, where progressive endothelial injury, microvascular rarefaction, fibroatrophy, hypocellularity, impaired osteoblast function, and diminished bone remodeling may persist despite visible bleeding [18,21,23,24,25,32,33,34,35,36].

Clinical observations are consistent with, but do not establish, this hypothesis. Zaghi et al. reported that about one quarter of patients developed persistent ORN after extensive mandibular resection and microvascular free-flap reconstruction despite resection to bleeding bone [14]. Notably, recurrent disease also occurred in patients whose final histopathological examination demonstrated margins free of necrotic bone, indicating that postoperative progression could not be explained solely by persistence of grossly necrotic tissue [14]. These findings do not establish that bleeding-guided margin selection caused treatment failure. Postoperative outcome after advanced ORN surgery is multifactorial and may also be influenced by disease severity, infection, soft-tissue condition, fixation and flap-related factors, reconstructive complexity, and patient-specific determinants of healing. Rather, the observations illustrate that bleeding and histopathological margin status alone are insufficient to predict durable postoperative disease control.

This possibility may be particularly relevant in early-onset or actively progressive ORN. During this stage, radiation-induced tissue injury in adjacent mandibular bone may still be evolving rather than having reached a stable fibroatrophic endpoint. Consequently, residual bone within high-dose irradiated regions may retain sufficient perfusion to produce punctate bleeding while also exhibiting ongoing endothelial dysfunction, progressive fibrosis, impaired cellular repopulation, and diminished regenerative reserve [18,21,23,24,25,32,33,34,35,36]. Such biological alterations may contribute to postoperative vulnerability, but they represent only one component of the multifactorial processes underlying delayed osteolysis, impaired union, fistula formation, or recurrent ORN. Although this hypothesis has not yet been directly validated, it is biologically consistent with current concepts of radiation-induced tissue injury and warrants prospective investigation.

Within this context, delayed postoperative failure should not be interpreted as evidence that bleeding-guided resection was inadequate. Rather, when failure occurs despite apparently viable margins, residual radiation-related tissue vulnerability may represent one of several contributing factors that conventional intraoperative assessment does not fully characterize. The DISM framework therefore proposes interpreting punctate marrow bleeding alongside patient-specific radiation dose distribution and other anatomical, radiological, host-related, and reconstructive determinants of postoperative healing, particularly when osteotomy margins lie within substantially irradiated regions [14,26,27].

### 6.4. Reframing the Surgical Endpoint Under the DISM Framework

The preceding evidence suggests that the conventional understanding of a successful mandibular osteotomy margin warrants re-evaluation. Although punctate marrow bleeding remains a useful intraoperative marker of residual perfusion, it should not be considered the exclusive determinant of biological suitability for long-term healing. Rather, assessment of the surgical endpoint should recognize that postoperative outcome reflects interactions among local tissue condition, prior radiation exposure, disease characteristics, host-related factors, and reconstructive variables, with intraoperative perfusion representing one component of this broader context.

**Figure 2:**
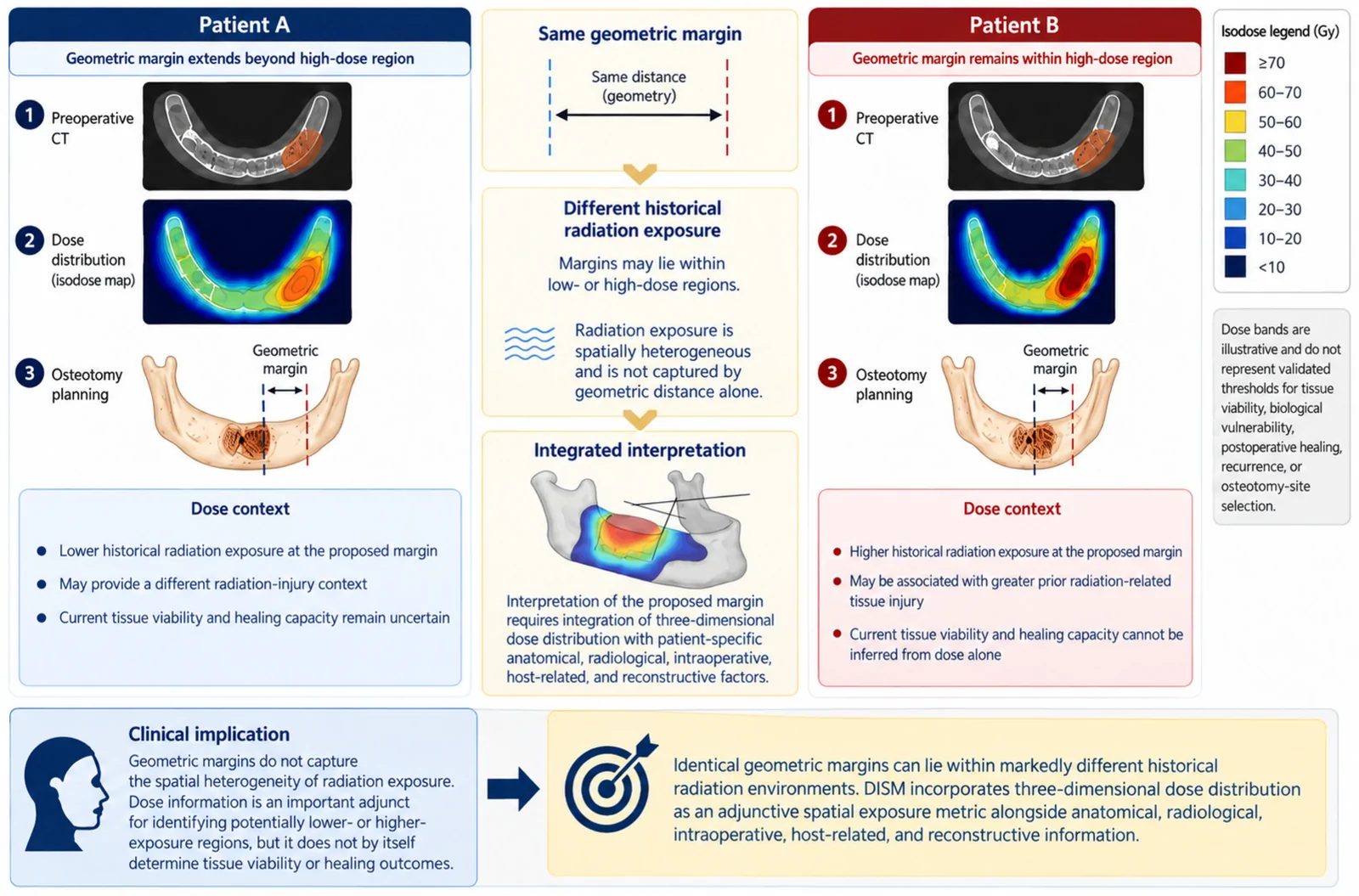
Identical Geometric Margins May Differ in Historical Radiation Exposure in Mandibular Osteoradionecrosis. Conceptual illustration showing that two geometrically comparable osteotomy margins may lie within substantially different regions of prior mandibular radiation exposure. The displayed dose ranges are illustrative and do not represent validated thresholds for tissue viability, biological vulnerability, postoperative healing, recurrence, or osteotomy-site selection. The Dose-Informed Surgical Margin (DISM) framework proposes interpreting patient-specific radiation dose distribution as an adjunctive spatial exposure metric alongside anatomical, radiological, intraoperative, host-related, and reconstructive considerations.

Within the DISM framework, mandibular resection is therefore not conceptualized simply as progression to the first bleeding bone. Instead, candidate osteotomy sites are evaluated through combined anatomical, radiological, intraoperative, dosimetric, and patient-related information. This approach does not imply that a particular combination of findings guarantees durable healing, but seeks to avoid reliance on any single surrogate of tissue viability. A candidate osteotomy margin may therefore warrant broader evaluation even when the exposed bone appears macroscopically viable and demonstrates punctate bleeding, particularly when substantial prior radiation exposure or other adverse biological or clinical factors are present [14,26,27]. Consequently, identical geometric osteotomy margins may correspond to markedly different historical radiation exposures, a distinction that geometric distance alone does not capture (Figure 2).

Accordingly, DISM does not aim to replace established intraoperative assessment methods or diminish the clinical value of punctate bleeding. Rather, it proposes integrating conventional surgical observations with patient-specific RT dosimetry, preoperative imaging, host-related factors, and reconstructive considerations to support individualized decision-making. Within this multidimensional framework, punctate bleeding is one element among several and should not be interpreted as an independent determinant of postoperative outcome.

DISM should currently be regarded as a hypothesis-generating conceptual framework rather than a validated surgical protocol. The proposed approach requires prospective evaluation, including correlation of RT dose distributions with histopathological findings, postoperative healing trajectories, recurrence patterns, and long-term reconstructive outcomes. Until such evidence is available, DISM should be understood as a biologically informed adjunct to established surgical judgment, not a replacement for conventional practice.

## 7. Potential Clinical Translation of DISM: From Radiotherapy Planning to the Operating Room

Although the DISM remains a conceptual and hypothesis-generating framework, advances in RT planning systems, multimodality imaging, image registration, and virtual surgical planning have created the technical foundation for its potential clinical application. Patient-specific RT dose distributions can now be retrieved, co-registered with contemporary imaging datasets, and incorporated into three-dimensional surgical planning platforms, enabling visualization of the spatial relationship between irradiated bone and planned osteotomy sites [26,27].

Within this context, radiation dosimetry should be regarded not as an isolated determinant of surgical decision-making, but as an additional source of biological information that complements conventional clinical, radiological, and intraoperative assessments. The primary aim of DISM is not to establish universal dose thresholds for mandibular resection, but to support individualized surgical planning by integrating patient-specific radiation exposure, anatomical findings, and other determinants of tissue competence.

The following sections outline a potential workflow for incorporating DISM into mandibular osteotomy planning, including retrieval and registration of RT data, integration with multimodality imaging and virtual surgical planning, multidimensional assessment of biological and anatomical variables, and use of DISM as an adjunctive decision-support framework rather than a replacement for established surgical judgment.

### 7.1. Retrieval and Registration of Radiotherapy Data

A foundational requirement for applying the DISM framework is retrieval of the original RT treatment plan, including the three-dimensional dose distribution, structure sets, and planning computed tomography (CT) datasets. When available, these data can be exported in Digital Imaging and Communications in Medicine (DICOM) format, providing the spatial information required for downstream registration and surgical planning [26,27]. In patients treated many years earlier, however, complete DICOM-RT datasets may be unavailable, incomplete, or incompatible with contemporary planning platforms; in such cases, reliable spatial reconstruction of historical dose may not be feasible.

Once retrieved, RT planning data can be co-registered with contemporary imaging studies obtained during evaluation of mandibular ORN, such as contrast-enhanced CT, cone-beam CT (CBCT), or magnetic resonance imaging (MRI), depending on the clinical context. Registration is intended to approximate the spatial relationship between historical radiation dose distribution and the current anatomy, allowing proposed osteotomy sites to be evaluated within their estimated prior radiation environment rather than on anatomical landmarks alone [26,27].

Registration accuracy may be substantially affected by anatomical changes occurring between the original planning CT and contemporary imaging. These may include tumor regression, prior surgery, fibrosis and soft-tissue remodeling, dental extraction or restoration, loss of teeth used as spatial landmarks, progressive ORN-related osteolysis, pathological fracture, mandibular deformation, segmental bone loss, and reconstructive procedures. Fixation hardware and dental materials may further degrade image quality through metal artifact and obscure otherwise useful anatomical landmarks. Consequently, rigid registration may be inadequate when major structural change has occurred, whereas deformable registration introduces its own uncertainty when anatomy has been substantially altered or tissue correspondence is no longer preserved. Registration should therefore be assessed using stable osseous landmarks and, where appropriate, complementary registration strategies rather than assumed to be geometrically exact [26,27].

Importantly, registration uncertainty propagates directly to uncertainty in the radiation dose assigned to a proposed osteotomy site. Even a modest spatial mismatch may be clinically relevant in regions with steep dose gradients, where small positional errors can materially alter the estimated local dose. Dosimetric information should therefore not be used to discriminate among candidate osteotomy sites when registration uncertainty is sufficiently large to make their relative dose exposure unreliable. In such circumstances, the DISM framework should defer to conventional anatomical, radiological, intraoperative, and reconstructive considerations rather than create false precision from uncertain dose mapping.

Reirradiation introduces an additional layer of complexity because a single historical RT plan no longer represents the total spatial exposure of the mandible. Where multiple RT courses have been delivered, meaningful interpretation may require registration of all available dose distributions and, when technically appropriate, cumulative dose reconstruction that accounts for differences in fractionation and spatial anatomy between treatment courses. If reliable dose accumulation cannot be achieved, the resulting exposure estimate should be regarded as uncertain and should not be treated as a quantitative basis for osteotomy-site selection.

Thus, registration should be regarded as an uncertainty-sensitive reconstruction of prior spatial radiation exposure rather than an exact recovery of the dose received by the current mandibular anatomy. Its value within DISM depends on the availability and quality of historical RT data, the degree of anatomical preservation, and the demonstrated reliability of the registration itself.

### 7.2. Integration with Multimodality Imaging and Virtual Surgical Planning

After image registration, patient-specific RT dose distributions may be incorporated with multimodality imaging and virtual surgical planning (VSP) platforms, supporting a three-dimensional representation of the irradiated mandible. Superimposing historical isodose distributions onto current anatomical datasets allows concurrent visualization of mandibular damage, adjacent soft tissue, and the spatial distribution of prior radiation exposure during preoperative planning [26,27].

Integrating RT dosimetry with contrast-enhanced CT, CBCT, and MRI leverages the complementary strengths of each modality to inform surgical decision-making. CT and CBCT provide high-resolution evaluation of cortical integrity, osseous architecture, and mandibular morphology, whereas MRI further characterizes bone marrow status and adjacent soft-tissue pathology. Interpreted alongside three-dimensional dose distributions, these imaging modalities may support a more comprehensive assessment of the biological context at the proposed osteotomy site [26,27].

Virtual surgical planning allows proposed osteotomy lines to be evaluated in relation to both anatomical structures and local radiation dose distribution before surgery. This approach moves beyond reliance on geometric distance from the visible margin of ORN, enabling assessment of whether a planned osteotomy traverses relatively lower- or higher-dose regions of the mandible. Importantly, this technique is intended to supplement, not replace, established anatomical planning or oncological principles.

Currently, evidence supporting dose-integrated virtual surgical planning largely comes from feasibility studies and preliminary clinical experience. Nonetheless, these technologies provide a practical basis for integrating the DISM concept into clinical workflows. Prospective investigations are needed to determine whether including patient-specific dosimetry in virtual surgical planning improves healing, reduces recurrence, or enhances reconstructive outcomes [26,27].

### 7.3. Multidimensional Osteotomy Planning

The DISM framework avoids relying on a single imaging modality or dosimetric parameter to determine mandibular osteotomy. Instead, it uses a multidimensional approach in which radiation dosimetry is interpreted alongside anatomical, radiological, intraoperative, clinical, and reconstructive findings to inform individualized surgical planning [14,26,27].

Within this framework, assessment of a candidate osteotomy site integrates several complementary domains: the macroscopic extent of ORN, radiological evidence of cortical and medullary bone destruction, spatial distribution of prior radiation dose, anticipated tissue condition, intraoperative assessment of bone perfusion, patient-specific biological attributes relevant to healing, and reconstructive feasibility. Synthesizing these variables may provide a more comprehensive assessment of the residual mandible than reliance on any single parameter [14,15,26,27].

When these domains are concordant, their combined interpretation may strengthen confidence in the suitability or unsuitability of a candidate osteotomy site. When they are discordant, however, DISM does not assign automatic priority to radiation dose or to any other single variable. Findings that directly indicate established disease, structural unsuitability, surgical safety concerns, or reconstructive infeasibility should not be overridden by a more favorable dose distribution. Conversely, a higher historical radiation exposure should not, by itself, mandate extension of the osteotomy when anatomical, radiological, intraoperative, and reconstructive findings otherwise support preservation of the segment. In such situations, dosimetry functions as a modifying source of spatial exposure information rather than as a prescriptive criterion.

The influence of dosimetry should also depend on the reliability of the underlying dose reconstruction. When historical RT data are incomplete or registration uncertainty prevents confident assignment of local dose, dosimetric information should be down-weighted or excluded from margin discrimination, and planning should rely primarily on established anatomical, radiological, intraoperative, and reconstructive considerations. When dose mapping is technically reliable but conflicts with other findings, the discordance itself should prompt multidisciplinary review rather than automatic selection of either the lower-dose or higher-dose site.

In this context, radiation dosimetry functions as a modifier of anatomical interpretation rather than an independent determinant. Two anatomically similar osteotomy sites may have substantially different historical radiation exposures and may therefore differ in their radiation-related tissue context. Conversely, preservation of anatomical structure alone does not necessarily establish sufficient regenerative potential, particularly where surrounding bone has sustained substantial cumulative radiation exposure [18,21,23,24,25]. DISM is not designed to replace established reconstructive principles or surgical judgment. Rather, the final osteotomy margin remains a multidisciplinary decision integrating disease extent, imaging characteristics, intraoperative findings, radiation history, patient-related factors, and reconstructive requirements.

### 7.4. Host Factors and Reconstructive Considerations

While local radiation dose offers insight into the biological status of irradiated mandibular bone, it does not solely determine postoperative healing or the likelihood of recurrent ORN. Patient-specific factors shape clinical outcomes by influencing vascular integrity, immune function, tissue oxygenation, and regenerative capacity. As a result, individuals with similar mandibular dose distributions may exhibit markedly different healing trajectories after comparable surgical interventions [1,2,3,4,18,23,24,25].

Host-related variables—including age, smoking status, diabetes mellitus, nutritional reserve, anemia, systemic inflammation, oral hygiene, periodontal disease, prior dentoalveolar trauma, and other comorbidities—can affect tissue resilience and wound healing after mandibular resection [1,2,3,4,18,23,24,25]. Similarly, treatment-related factors, such as radiation technique, fractionation, concomitant systemic therapy, previous surgical interventions, and timing and extent of reconstruction, may further shape the biological response of irradiated tissues and influence postoperative outcomes [1,2,3,4,18,23,24,25].

These considerations indicate that radiation dose is most appropriately interpreted within a broader clinical context. In the DISM framework, patient-specific dosimetry is one element of a comprehensive biological assessment, in which host vulnerability and reconstructive variables provide additional perspective on the residual mandible’s capacity to sustain durable healing. Consequently, osteotomy margin selection should be individualized, integrating local tissue characteristics and the patient’s overall biological resilience.

From a reconstructive standpoint, DISM is designed to complement, not replace, established principles of mandibular reconstruction. The choice of reconstructive technique, flap design, fixation strategy, and soft-tissue management should remain guided by disease extent, anticipated functional needs, and surgical expertise. In this context, patient-specific radiation dosimetry may offer supplementary insight into the biological environment for reconstruction, supporting informed multidisciplinary planning while preserving established reconstructive standards.

### 7.5. DISM as an Adjunctive Decision-Support Framework

The DISM framework is designed to serve as an adjunctive decision-support framework, not as an autonomous method for determining mandibular osteotomy margins. Its principal aim is to provide supplementary spatial information derived from patient-specific RT dosimetry, thereby complementing conventional anatomical, radiological, intraoperative, host-related, and reconstructive assessment while preserving established surgical judgment [14,26,27].

Within this framework, radiation dose is considered one element of a multidimensional assessment rather than an independent determinant of surgical decision-making. Osteotomy-margin selection therefore continues to rely on multidisciplinary integration of clinical examination, imaging findings, intraoperative observations, reconstructive requirements, and patient-specific biological variables. No single parameter is assigned universal priority across all clinical scenarios.

When individual domains are discordant, the framework does not prescribe automatic selection of the lower-dose site or extension of resection solely because a candidate margin lies within a higher-dose region. Findings indicating established disease, structural unsuitability, surgical risk, or reconstructive infeasibility retain priority over favorable dosimetry. Conversely, when anatomical, radiological, intraoperative, and reconstructive findings support preservation, historical radiation exposure alone does not mandate a more extensive osteotomy. The influence of dosimetry should further be reduced when registration or dose-assignment uncertainty is substantial.

DISM therefore provides additional context when conventional assessment leaves uncertainty regarding the relative suitability of candidate osteotomy sites, while preserving the primacy of established surgical and reconstructive principles. In this regard, the framework is intended to support clinical reasoning rather than supplant it.

Future prospective investigations are needed to establish the most effective way to incorporate DISM into multidisciplinary head and neck practice. Correlating RT dose distributions with histopathological findings, intraoperative observations, postoperative healing, recurrence patterns, functional outcomes, and reconstructive success will be essential to determine its clinical validity and delineate its role in surgical care.

## 8. Future Directions and Research Priorities

The DISM framework is proposed as a biologically informed, hypothesis-generating approach intended to complement conventional surgical planning in mandibular ORN. While its rationale is grounded in established principles of radiation biology, mandibular dosimetry, imaging, and surgical management, the framework remains unvalidated in prospective clinical studies. Accordingly, its application in routine practice should be considered investigational until further research defines its clinical utility.

Further research should prioritize validating the biological assumptions underpinning DISM, assessing its impact on surgical and reconstructive outcomes, refining techniques for integrating RT dosimetry into clinical workflows, and identifying patient-specific factors influencing tissue competence after irradiation. The following sections outline key research priorities needed to translate DISM from a conceptual proposal into an evidence-based adjunct in individualized mandibular osteotomy planning. Table 2 summarizes the major research priorities and proposed translational pathway for clinical validation of the DISM framework.

### 8.1. Biological Validation of the DISM Framework

A principal research priority for the DISM framework is to determine whether spatial variation in historical mandibular radiation exposure is associated with measurable differences in the biological state and healing capacity of residual irradiated bone. Although numerous studies have reported associations between radiation dose and ORN risk, it remains uncertain whether local dose at a proposed osteotomy site reliably corresponds to current perfusion, cellular viability, remodeling activity, regenerative capacity, or subsequent healing [3,5,6,7,8,23,24,25,26,27].

Future investigations should integrate RT dosimetry with histopathological, molecular, and imaging analyses of resected mandibular specimens. Correlating local radiation dose with parameters such as vascular density, endothelial integrity, osteocyte and osteoblast viability, bone remodeling activity, fibrosis, inflammatory cell infiltration, and additional markers of tissue repair may help determine how local radiation dose relates to specific biological domains, including vascular integrity, cellular viability, remodeling activity, and reparative capacity [15,18,21,23,24,25,41]. Such studies may also clarify whether a transitional zone of biologically compromised, yet not overtly necrotic, bone exists beyond the macroscopically apparent ORN margins, as proposed by the biological penumbra concept [15,23,24,25].

Advanced imaging modalities and functional biomarkers may offer complementary strategies for validating this hypothesis. Correlating dose distributions with functional imaging, quantitative MRI, nuclear medicine techniques, or emerging molecular biomarkers could improve understanding of the spatial heterogeneity of radiation-induced mandibular injury and help identify imaging surrogates for tissue competence [15,26,27,43].

Collectively, these lines of inquiry will be important for determining whether objective experimental and clinical evidence substantiates the biological assumptions underlying DISM. Such validation would provide a necessary foundation for subsequent clinical evaluation of dose-informed mandibular osteotomy planning [14,15,26,27,43].

### 8.2. Clinical Validation and Outcome Assessment

If biologically validated, the next essential step is to determine whether DISM-informed planning is associated with clinically meaningful advantages over conventional margin assessment. Prospective studies should evaluate predefined outcomes across complementary domains rather than relying on a single endpoint. Disease-control and healing outcomes should include complete mucosal healing within a prespecified interval, persistent or recurrent ORN, fistula formation, clinically significant infection, and time to disease progression or recurrence. Reconstructive outcomes should include osseous union or nonunion, plate exposure, flap-related complications, reconstructive failure, and the need for revision surgery [14,15,26,27,43].

Outcome definitions and assessment time points should be standardized prospectively. Early postoperative healing and later disease control should be evaluated separately because delayed complications may emerge months or years after an initially successful reconstruction. Where applicable, outcomes should be reported longitudinally and with sufficient follow-up to distinguish persistent disease, delayed recurrence, and late reconstructive failure.

Clinical validation should also incorporate functional and patient-reported outcomes, including pain, mastication, speech, swallowing, mandibular continuity, ability to undergo dental rehabilitation, quality of life, and patient-reported functional recovery. These outcomes are necessary to determine whether a change in osteotomy planning provides meaningful benefit beyond radiological or surgical disease control.

Appropriate study designs may include prospective observational cohorts, matched comparative studies, and ultimately multicenter trials comparing conventional planning with DISM-informed approaches. Given the heterogeneity and relative infrequency of ORN, multicenter collaboration will likely be required to achieve adequate sample size, capture variation in tumor sites and RT techniques, and assess reproducibility across institutions.

Clinical validation should avoid reducing DISM to a single-dose cutoff. Rather, studies should evaluate radiation dose within the same multidimensional framework used for planning, incorporating anatomical disease extent, imaging findings, intraoperative assessment, host vulnerability, reconstructive variables, and uncertainty in dose localization. Such studies should determine not only whether DISM is associated with improved outcomes, but also which outcome domains, patient subgroups, and clinical scenarios derive meaningful incremental benefit from incorporating historical dosimetry.

### 8.3. Imaging, Functional Assessment, and Digital Surgery

Advancing the DISM framework will likely require integrating RT dosimetry with advanced imaging modalities and digital surgical technologies. Existing evidence indicates that conventional anatomical imaging may be insufficient to comprehensively characterize the biological extent of mandibular ORN, particularly when functional alterations precede detectable structural changes. Consequently, future research should assess whether combining RT dose mapping with CT, CBCT, MRI, functional imaging, and virtual surgical planning improves evaluation of residual mandibular tissue competence [15,26,27,43].

Functional imaging represents a potentially valuable adjunct in this context. Techniques such as SPECT-CT offer insights into bone turnover, inflammatory processes, and regional biological activity, complementing both radiation dose distribution and morphological imaging. Recent studies using SPECT-CT to determine bony resection margins in inflammatory jaw pathologies suggest that functional imaging may help define biologically appropriate margins and inform reconstructive planning, although its specific utility in mandibular ORN warrants further investigation [43].

Digital surgical platforms are likely to facilitate DISM implementation. Integration of three-dimensional dose overlays, deformable image registration, automated mandibular segmentation, virtual osteotomy simulation, patient-specific cutting guides, intraoperative navigation, and augmented reality visualization could enable direct incorporation of radiation dose information into operative planning and execution. Nevertheless, rigorous evaluation of these tools is needed to assess registration accuracy, workflow feasibility, reproducibility, cost, and clinical impact before routine adoption [25,26,43].

Ultimately, imaging and digital surgery should be regarded as complementary modalities for advancing DISM from conceptual framework to clinical practice. Their value will be determined not only by technical feasibility but also by demonstrated improvements in biological margin assessment, reduction of postoperative complications, and enhanced support for durable functional reconstruction.

### 8.4. Toward Personalized ORN Surgery

Advancing the long-term development of DISM will likely require a shift from dose visualization alone toward a personalized paradigm for mandibular ORN surgery. Given that radiation-induced bone injury is influenced by host vulnerability, dental status, systemic inflammation, nutritional reserve, vascular health, reconstructive variables, and the temporal evolution of radiation injury, future frameworks should incorporate dosimetric, imaging, clinical, and biological data into individualized risk-assessment models [1,2,3,4,18,23,24,25,32,33,34,35,36,41]. A conceptually similar persistence of treatment-related vulnerability has been reported in MRONJ, although MRONJ and ORN are etiologically distinct; such observations reinforce the broader rationale for individualized, multidimensional assessment rather than reliance on a single contemporary surrogate of tissue competence [42].

Beyond interpatient biological variability, the stage and temporal evolution of ORN itself may influence the clinical utility of DISM. Because radiation-induced fibroatrophic injury evolves over time, incorporating patient-specific dosimetry may be particularly informative in relatively early or actively progressive disease, whereas its incremental contribution in long-standing stable ORN remains to be determined [14,21,23,24,25,44].

Within this context, RT dose distribution may serve as the spatial foundation for more comprehensive predictive models. Variables such as mandibular subsite, cortical versus cancellous bone involvement, dental extraction history, periodontal status, hemoglobin concentration, inflammatory or immune-nutritional biomarkers, comorbidities, smoking status, temporal disease characteristics, and planned reconstructive strategy may further refine the estimation of residual mandibular competence and postoperative healing potential.

Machine-learning and radiomics-based methodologies may ultimately facilitate this process by elucidating complex interactions among dosimetric variables, imaging phenotypes, host characteristics, temporal disease patterns, and clinical outcomes. Nonetheless, responsibly integrating such models into surgical decision-making will require large multicenter datasets, standardized imaging and dosimetric protocols, transparent reporting, external validation, and clinically interpretable results.

The overarching objective of personalized ORN surgery should extend beyond removal of necrotic bone to selecting residual mandibular segments with the highest likelihood of durable healing and functional reconstruction tailored to the individual patient. DISM may offer a conceptual basis for this transition by integrating radiation dose distribution with individualized biological risk assessment.

### 8.5. Limitations of the Proposed Framework

The DISM framework presents several notable limitations. Primarily, it is a conceptual, hypothesis-generating model synthesized from existing radiobiological, dosimetric, imaging, histopathological, and surgical literature, rather than informed by prospective clinical validation. As such, DISM should not be regarded as a validated surgical protocol or as substantiation for modifications to current standards of care.

Second, while radiation dose is a well-established contributor to ORN risk, it constitutes only one facet of the multifactorial biological processes underlying tissue injury and repair. The direct relationship between local radiation dose and the biological competence of residual mandibular bone remains unestablished. Accordingly, dose thresholds derived from studies of ORN development should not be interpreted as validated thresholds for osteotomy-site selection or postoperative outcome prediction. Individual variability in host factors, vascular integrity, systemic inflammation, nutritional status, dental health, and reconstructive considerations may significantly influence tissue response following irradiation [1,2,3,4,18,23,24,25,32,33,34,35,36].

Third, the DISM framework does not endorse universal radiation dose thresholds for determining mandibular osteotomy margins. Radiation-induced tissue injury occurs along a biological continuum, and identical dose distributions may yield disparate clinical outcomes in different individuals. Consequently, radiation dosimetry is best interpreted as one element within a multidimensional biological assessment, rather than an independent determinant of surgical decision-making [14,15,26,27].

**Figure 3:**
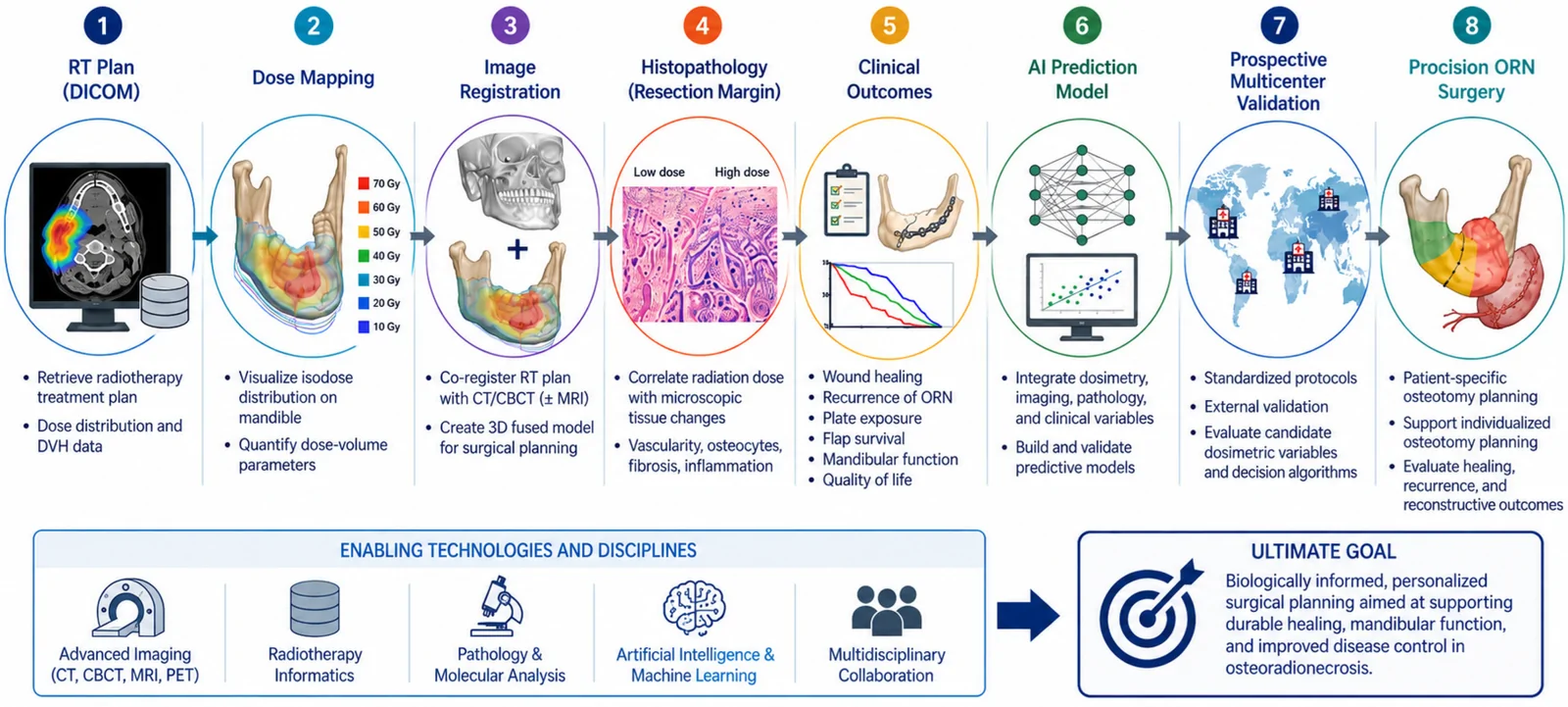
Proposed Roadmap for Clinical Validation and Translation of the Dose-Informed Surgical Margin (DISM) Framework. The roadmap illustrates the progression from retrieving radiotherapy planning data and mapping patient-specific dose to image registration, biological validation, clinical outcome assessment, predictive model development, multicenter validation, and eventual integration into personalized mandibular osteotomy planning. Abbreviations: AI, artificial intelligence; CBCT, cone-beam computed tomography; CT, computed tomography; DICOM, Digital Imaging and Communications in Medicine; DVH, dose–volume histogram; MRI, magnetic resonance imaging; ORN, osteoradionecrosis; PET, positron emission tomography; RT, radiotherapy.

Finally, successful implementation of DISM relies on access to complete historical RT datasets, sufficiently reliable registration between historical and contemporary anatomy, multidisciplinary collaboration, and suitable digital planning infrastructure—requirements that may be compromised by unavailable DICOM-RT data, major anatomical change, metal artifact, or prior reirradiation. When these factors prevent reliable localization or accumulation of prior dose, the uncertainty should limit the influence of dosimetry on osteotomy-site selection. Future prospective research incorporating standardized dosimetric protocols, biological validation, and multicenter collaboration will be required to establish the clinical utility, reproducibility, and generalizability of the DISM framework. Figure 3 illustrates the proposed translational pathway from biological validation to clinical implementation of the DISM framework.

## 9. Conclusions

Mandibular ORN remains a persistent and complex late complication of head and neck RT. Determination of an appropriate osteotomy margin is still predominantly informed by anatomical findings, intraoperative assessment, and surgical judgment. However, accumulating evidence indicates that radiation-induced mandibular injury is biologically heterogeneous and spatially variable. Consequently, visible necrosis, millimetric distance, and punctate marrow bleeding may not fully reflect the biological competence of residual irradiated bone.

The DISM framework is introduced as a biologically informed, hypothesis-generating approach that integrates patient-specific RT dosimetry with conventional anatomical, radiological, intraoperative, and host-related variables to inform individualized mandibular osteotomy planning. DISM is not intended to replace established surgical principles, but rather to add a complementary biological perspective for interpreting tissue competence and selecting osteotomy margins.

At present, DISM should be regarded as a conceptual framework rather than a validated surgical strategy. Prospective biological and clinical validation is required before routine adoption. If validated, DISM may facilitate a shift from predominantly anatomical definitions of mandibular osteotomy toward biologically informed, patient-specific surgical planning, offering a revised conceptual paradigm for the surgical management of mandibular ORN.

## Figures and Tables

**Table 1:** Evidence Domains and Gaps Relevant to the DISM Framework.

Evidence Domain	Key Finding	Relevance to DISM and Evidence Gap	Key References
Mandibular dosimetry and ORN risk	Higher mandibular dose-volume exposure is consistently associated with increased ORN risk	Establishes the association between mandibular radiation exposure and ORN development; provides an indirect biological rationale for DISM but does not validate osteotomy-site dose as a predictor of postoperative outcome	[3,5,6,7,8,32,33,34,35,36]
Isodose-informed surgical planning	Patient-specific isodose visualization can be integrated into mandibular ORN surgical planning.	Provides direct evidence of technical feasibility and preliminary surgical applicability, but current evidence is insufficient to establish validated osteotomy-site dose criteria or outcome thresholds	[26,27]
Postoperative progression despite bleeding-guided margins	ORN progression may occur despite resection to bleeding bone and histologically negative margins	Indicates that bleeding and histopathological margin status alone do not ensure durable postoperative disease control; this observation does not establish that the margin-assessment method caused treatment failure	[14]
Histopathological heterogeneity	Cortical and cancellous bone may show heterogeneous necrotic and viable changes at resection margins	Provides a biological rationale for the hypothesized biological penumbra and for spatially variable tissue competence	[15,16]
Functional imaging and biological margin assessment	SPECT-CT-guided margin determination may help identify biologically adequate bony margins in inflammatory jaw pathologies	Supports the broader principle that functional/biological data can complement anatomical margin planning	[43]
Dose–timing interaction	Mandibular radiation dose modifies the relationship between post-treatment dental extraction timing and ORN risk	Supports the concept that radiation dose interacts with temporal and traumatic factors to shape tissue vulnerability	[44]
Host vulnerability and multifactorial risk	ORN risk and healing outcomes are influenced by dental status, systemic factors, comorbidities, inflammation, and tissue repair capacity	Reinforces DISM as a multidimensional framework rather than a dose-only model	[1,2,3,4,13,18,23,24,25]

Abbreviations: DISM, Dose-Informed Surgical Margin; ORN, osteoradionecrosis; SPECT-CT, single-photon emission computed tomography/computed tomography. Note: This table summarizes selected evidence domains relevant to the DISM concept and their principal evidentiary limitations. The evidence is heterogeneous and includes direct, indirect, translational, and hypothesis-generating observations; the table is not intended as a systematic evidence synthesis, evidence hierarchy, or demonstration of clinical validation of DISM.

**Table 2:** Proposed Decision-Support Workflow for the Dose-Informed Surgical Margin (DISM) Framework.

Workflow Step	Required Data	Purpose	Current Evidence Level	Validation Need
Retrieval of radiotherapy data	Original RT plan, DICOM dose, structure sets, planning CT	Reconstruct the spatial history of mandibular radiation exposure	Technical feasibility shown in early studies	Standardized data retrieval protocols
Image registration	Planning CT co-registered with current CT, CBCT, or MRI	Align historical dose distribution with current ORN anatomy	Feasible, but registration and dose-assignment uncertainty remain	Registration accuracy, dose-assignment uncertainty, and reproducibility testing
Isodose visualization in VSP	Registered dose map integrated into 3D surgical planning platform	Visualize planned osteotomy lines relative to local radiation dose	Supported by feasibility and early clinical reports	Prospective correlation with predefined healing, disease-control, and reconstructive outcomes
Anatomical disease assessment	CT/CBCT/MRI, clinical examination, intraoperative findings	Define gross disease extent, cortical destruction, medullary involvement, and soft-tissue status	Established component of ORN management	Standardized multimodality assessment criteria
Intraoperative viability assessment	Visual inspection, tactile assessment, punctate bleeding, perfusion adjuncts if available	Estimate immediate residual perfusion and macroscopic tissue viability	Widely used but biologically incomplete	Correlation with long-term healing and histopathology
Host vulnerability assessment	Smoking, diabetes, anemia, nutrition, inflammation, dental/periodontal status, extraction history	Contextualize dose effects within patient-specific repair capacity	Supported by multifactorial ORN risk literature	Integrated predictive modeling
Conflict resolution and weighting of inputs	Concordance or discordance among anatomical, radiological, intraoperative, dosimetric, host-related, and reconstructive findings; reliability of dose registration	Determine the appropriate influence of each domain when findings conflict. Established disease, surgical safety, and reconstructive feasibility are not overridden by favorable dosimetry; higher dose alone does not mandate wider resection. Unreliable dose mapping is down-weighted or excluded from margin discrimination	Conceptual DISM decision rule; not clinically validated	Prospective testing of decision consistency, reproducibility, and clinical outcomes
Multidisciplinary margin selection	Integrated dosimetry, imaging, intraoperative findings, host factors, reconstructive plan, and assessment of uncertainty	Select the candidate osteotomy site through integrated interpretation of all domains after resolving discordant or unreliable inputs	Conceptual DISM framework	Prospective validation using predefined healing, disease-control, reconstructive, functional, and patient-reported outcomes

Abbreviations: CBCT, cone-beam computed tomography; CT, computed tomography; DISM, Dose-Informed Surgical Margin; DICOM, Digital Imaging and Communications in Medicine; MRI, magnetic resonance imaging; ORN, osteoradionecrosis; RT, radiotherapy; VSP, virtual surgical planning. Note: This workflow is proposed as a conceptual decision-support framework and does not define validated dose thresholds, prescribe a fixed hierarchy among all inputs, or replace established surgical judgment. Where inputs conflict, established disease, surgical safety, and reconstructive feasibility retain priority, while the influence of dosimetry depends on the reliability of spatial dose reconstruction. Proposed clinical validation outcomes are outlined in Section 8.2 and should be prespecified and assessed longitudinally.

## Data Availability

No new data were created or analyzed in this study.
